# Traumatic brain injury in children: 18 years of management

**DOI:** 10.11604/pamj.2020.37.235.23400

**Published:** 2020-11-13

**Authors:** Romuald Kouitcheu, Moussa Diallo, Alban Mbende, Aïcha Pape, Ernest Sugewe, Guy Varlet

**Affiliations:** 1 Neurosurgery Department, University Hospital Center of Yopougon, Abidjan, Ivory Coast,; 2 Neurosurgery Department, University Hospital Center of Gabriel Touré, Bamako, Mali,; 3 Neurosurgery Department, King´s College Hospital National Health Service Trust, London, United Kingdom

**Keywords:** Brain injury, traumatic, child, Ivory Coast, surgery

## Abstract

Traumatic brain injury in children is a common cause of emergency department admission to our institution. The aim was to summarize the management of all head injuries in children. This was a retrospective, descriptive single center study performed in the Neurosurgery Department, University Hospital Center, Yopougon-Abidjan, Ivory Coast from January 2000 to December 2017. We included all patients less than 16-years-old admitted to the emergency department and all admitted in neurosurgery department for a traumatic brain injury with a cerebral tomodensitometry and/or a magnetic resonance imaging. 292 patients were admitted in neurosurgery department during the study period. The average age of our patients was 7.8 ± 0.80 years with a male predominance (64%). Road accidents were the main causes (78.7%) followed by falls. Brain trauma was mild in 53.8% of cases, moderate in 36.8% and severe in 9.4% of cases. Initial loss of consciousness and headache were the main reasons for admission to the emergency room after the injury with a proportion of 87.6%. The oedemato-haemorrhagic contusion was the most frequent lesion found in our patients with a frequency of 33.9%. The surgery was performed in 36.9% of cases. The overall mortality of patients in the study remains high with a proportion of 13.18%. Traumatic brain injuries in children had a high mortality rate in our practice. Specialized centers should be developed to optimize their care.

## Introduction

Traumatic brain injury (TBI) is the result of a direct or indirect mechanical aggression on the skull and underlying brain, immediately present disorders of consciousness translating diffuse or localized brain suffering ranging from obnubilation to coma. In developing countries, this condition has become more frequent with the upsurge of road accidents. The possibility of serious secondary complications, sometimes disabling sequelae, socio-economic fallout makes care difficult. TBI represent the first cause of admission to the pediatric emergencies of our institution. TBI constitute a real public health problem in developed countries and marked increase in underdeveloped countries. In the United States alone, an estimated 475,000 children aged 0-14 suffer a traumatic brain injury (TBI) each year. TBI results in more than 7000 deaths, 60,000 hospitalizations, and 600,000 emergency department visits annually among American children [1]. Similarly, TBI affects the pediatric population worldwide. Studies have shown that TBI contributes to more than half of pediatric injuries in Iran, around 20% of trauma emergency department admissions in India, and around 30% of pediatric injuries in Korea [2]. Furthermore, TBI affects more than 486 adolescents per 100,000 people per year in Australia and approximately 280 children out of 100,000 people in the United Kingdom [3]. Compared with their adult counterparts, children suffering head injury warrant particular concern given the developmental consequences of early brain damage. The aim of this study is to describe the epidemiological, clinical, therapeutic and evolution aspects of children traumatic brain injuries in the Neurosurgery Department, Yopougon Teaching Hospital, while underlining the difficulties of the adequate management of this affection.

## Methods

It was a retrospective, descriptive single center study performed in the Neurosurgery Department, University Hospital Center, of Yopougon-Abidjan, Ivory Coast over a period of 18 years ranging from January 2000 to December 2017.

**Inclusion criteria**: were included all children aged less than 16 years old admitted to pediatric emergencies of our institution then hospitalized in the neurosurgery department for a traumatic brain injury with a cerebral Computed tomography (CT) and/or an magnetic resonance imaging (MRI).

**Exclusion criteria:** were excluded all children aged less than 16 years old admitted during the study period from traumatic brain injury whose records were incomplete.

**Analyzed parameters:** the epidemiological, clinical, therapeutic and evolution aspects of children´s traumatic brain injuries in the Neurosurgery Department, Yopougon Teaching Hospital were analyzed. We report only the results of analysis of patients hospitalized in neurosurgery, while underlining the difficulties of the adequate management of this affection. The analysis and interpretation of these different criteria were made using EPIINFO software version 6.06 D. We used the following description tools: graphs and tables (value and percentage).

## Results

**Epidemiological criteria:** during the study period 2825 cases of traumatic brain injuries in children aged less than 16 years old admitted to pediatric emergencies of our institution; among them 1020 (36%) presented clinical abnormalities and/or imaging. 292 (10.34%) children were hospitalized in neurosurgery department.

**Figure 1:**
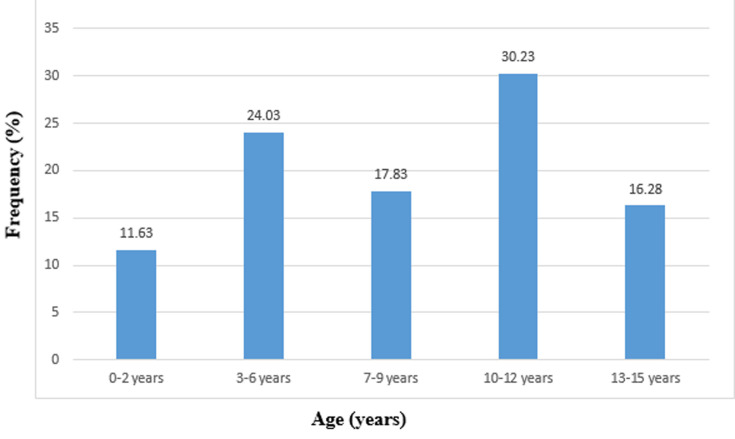
distribution of children by age

**Age:** the average age of our patients was 7.8 ± 0.80 years old with extremes (2 days-15 years). The most affected age group was (10-12) years (Figure 1).

**Sex:** our study population consisted of 187(64%) male and 105(34%) female. We observed a male predominance with a sex ratio1.8.

**Figure 2:**
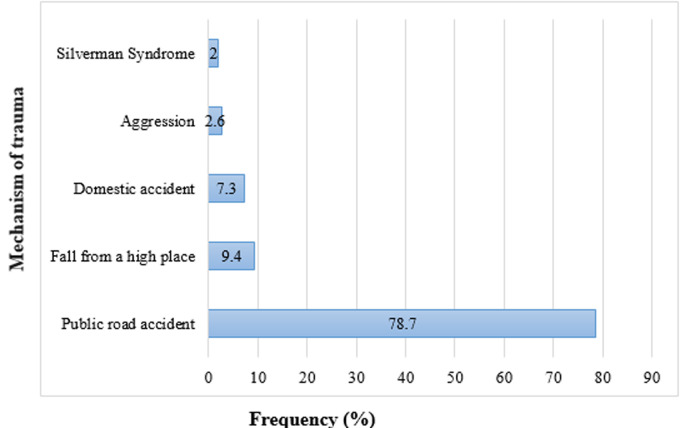
distribution of children by the mechanism of trauma

**Mechanism of trauma:** road accidents were the main causes (78.7%) followed by falls (Figure 2).

**Pre-hospital treatment:** one hundred and thirty-one (45%) had received medical treatment prior to admission. This treatment was indicated in 72 children: 12 had received an analgesic, 21 had venous access, 18 had received anticonvulsant treatment, 3 had oxygen therapy and 18 had received wound suture.

**Transfer to Yopougon university hospital:** transfer to Yopougon university hospital was done by vehicle in 45.14% of cases, motorcycle in 12.71% of cases, ambulance in 10% of cases and firefighters in 8% of cases. The mode of transport was not specified in 24.31% of the cases. The admission time was less than 24 hours in 70.20% of cases, between 24 hours and 72 hours in 20.38% of cases and more than 72 hours in 8.42% of cases.

### Clinical criteria

**Glasgow Coma Scale (GCS) and injury severity (Table 1)**: 157 children (53.8%) had mild TBI (15 ≤ GSC ≤ 12), 107 (36.8%) had moderate TBI (12 ≤ GCS ≤ 8) and 28 (9.4%) had severe TBI (GSC ≤ 8).

**Table 1:** distribution of children by Glasgow Coma Scale (GCS) and injury severity

Injury severity	GCS	Patients number	Frequency
Minimal	12-15	157	53.8%
Moderate	8-12	107	36.8%
Severe	< 8	28	9.4%

**Physical examination:** initial loss of consciousness and headache were the main reasons for admission to the emergency room after the injury with a proportion of 87.6% (Table 2).

**Table 2:** distribution of patients by clinical signs at admission

	Signs	Number/Frequency
Functionnal signs	**Loss of initial knowledge**	**256 (87.6%)**
	**Headache**	**231 (79.2%)**
	Convulsions	25.(19.4%)
	Vomiting	18.(14%)
	Pathological flows	21.(16.3%)
Examination of the encephalic extremity	**Scalps wounds**	**76.(59%)**
	Periorbital bruises	14 (11%)
	Brain materials issues	9 (70%)
Neurologic examination	Anisocoria	13 (10%)
	Motor Deficit	6 (5%)
Somatic examination	**Skin dermabrasions**	**106 (82%)**

### Paraclinical criteria

**Figure 3:**
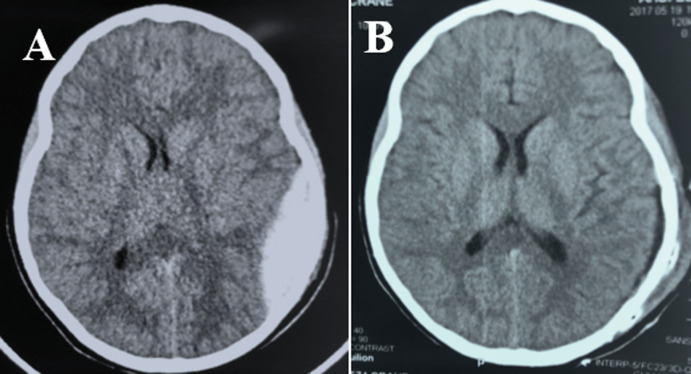
cerebral scan showing a left parietal-temporal extradural hematoma, pre-operative (A); postoperative (B)

**Anatomoclinical lesions:** of the 292 children 64 (22%) had a skull X-ray and all had a brain scan (Figure 3). The oedemato-haemorrhagic contusion was the most frequent lesion found in our patients with a frequency of 33.9%(Table 3).

**Table 3:** distribution of children on observed anatomoclinical lesions

Lesions	Number	Frequency (%)
Acute subdural hematoma	18	6.25
Oedemato-haemorrhagic contusion	99	33.9
cranio-cerebral wounds	22	7.5
Abs of parenchymal lesions	131	44.8
Intraparenchymal hematoma	9	3.12
Epidural hematoma	24	8.3
Simples fractures	15	5.2
Depressed skull fractures	73	25
Skull base fractures	24	8.3
Abs of bone lesions	102	34.9

**Lesions associated with brain trauma:** the limb trauma and cervical spinal trauma was the most frequent lesion associated found in our patients with a respectively frequency of 9.3% and 5.4% (Table 4).

**Table 4:** distribution of children by lesions associated with brain trauma

Associated lesions	Number	Frequency (%)
Limb trauma	27	9.3
Cervical spine trauma	16	5.4
Thoracic spine trauma	2	0.77
Rib fractures	2	1.55
Maxillo-facial trauma	7	2.32

**Therapeutic criteria:** (Table 5) shows the distribution of treatments used in these children. Of the children admitted, 108 (36.9%) required surgical treatment. Of the 108 children who had been operated on, 41 had extra-traumatic hematoma evacuation, 22 had a cranio-cerebral wound healing and 36 had an evacuation and 9 had a hematoma evacuation. Under dural acute with a decompressive flap. Operative follow-up was simple in 95 children and was complicated by pulmonary infections in 4 children and operative wound in 9 children.

**Table 5:** distribution of children according to the treatments used

Evolution		Number	Frequency (%)
Favorable	Without sequelae	229	78.42
	With sequelae	25	8.40
	Psychomotor	16	5.32
	Motor deficiency	9	3.08
Unfavorable/death		38	13.18
**Total**		**292**	**100**

**Evolution criteria:** the duration of hospitalization was 1 to 10 days for 190 (65%) children, 11 to 30 days for 73 (25%) children and more than 31 days for 29 (10%) children. The mean follow-up was 372 days (range 14 to 5460 days). The evolution was favorable in 253 (86.8%) children with sequelae in 25 children (16 psychomotor and 9 motor deficits). Eight traumatized head children died on admission before receiving care. The overall mortality was 13.18%, and 61.04% among the severe TBI (Table 5, Table 6). After the discharge, 65 of the 108 operated patients were seen again in the control clinic. Four children should have cranioplasty. The other children were lost to sight.

**Table 6:** distribution of children by evolution

Evolution		Number	Frequency (%)
Favorable	Without sequelae	229	78.42
	with sequelae	25	8.40
	Psychomotor	16	5.32
	Motor deficiency	9	3.08
Unfavorable/death		38	13.18
Total		292	100

Average decline: 372 days (range: 14 to 5460 days)

## Discussion

**Epidemiology:** this study made it possible to analyze the socio-demographic characteristics of children with traumatic brain injury, to identify the causes, and the technical difficulties related to their care in our hospital.

**Age:** the age group concerned is 1 to 15 years old with an average age of 7.80 ± 0.8 years. However, the age group of 10-12 is the most concerned. This slice is the most watched. Murgio [4] noted in his series that children from 0-4 years are the biggest victims with 55.2%. Max Decamps [5] found that the age group of 5-8 and 12-15 are more concerned. In our study, the average age of children was 7.8 ± 0.8 years; in Tunisia, Hassen *et al*. had a mean age of 5.9 ± 3.9 years [6], and Bahloul an average age of 7.54 ± 3.8 years [7], as well as Kpelao in Dakar: 7.5 years [8].

**Sex:** in our study, it was noted a male predominance with 64% against 36% for the female, i.e sex ratio of 1.8. A multi-center study conducted by Murgio and collaborators [4] in Brazil, France, Hong Kong and Spain out of a total of 2478 patients, also showed a male predominance with 60.9% compared to 39.1%, i.e sex ratio of 1.5. A distribution of 2 girls for five boys was noted by Max Decamps [5] in Senegal. Several studies [6-8] also observed a male predominance among children with head trauma as in our study. This predominance could be explained naturally because at the same age the boy is more turbulent than the girl. The second argument is customary because in Africa the little girl is often confined to the home learning domestic work while the boy enjoys greater freedom and therefore greater mobility that exposes to the brain-encephalic trauma.

**Mechanism of trauma:** the children´s cranio-encephalic traumas recognize several etiological circumstances, the most common of which is the road accident. This is clearly growing in our country. Road accidents were the main cause of head trauma in our study as in Dakar [8]. Indeed Decamps [5] noted that a rate of 43% of children in his series are victims of traffic accident in Senegal. Emanuelson *et al*. [9] meanwhile found 60% of cases. This trend has been described by other authors [10-12]. In Tunisia, Hassen *et al*. had observed a predominance of domestic accidents [6]. In our series, it was noted a rate of 78.8% of accident of the public road. This frequency is followed by that of domestic accidents with 16%. These domestic accidents are of various natures: it can be falls of its height, the height of a table, falls of the balcony or weak and poorly made buildings which fall on a child. This distribution shows us that, despite the greater importance of the car fleet, the cranio-encephalic traumas of the child have become more frequent in our country. It was also noted the scarcity of this scourge in the rural population compared to cities. This difference is easily explained by impoverishment, the concentration of the population in the cities and the lack of education of the children.

**Pre-hospital care:** in our series, one hundred and thirty-one children (45%) had received medical treatment before admission to the Yopougon university hospital. This treatment was specified in 72 children, but only 12 received an analgesic. The management of pain was not systematic in the peripheral hospitals in the management of traumatic brain injury, hence awareness is needed. In our study, the majority of transfers to the Yopougon university hospital were by means of private travel 45.14% of cases. In Senegal, the majority of transfers were made by the fire brigade or the emergency medical aid service [8]. The admission time was less than 24 hours in 70.20% of cases, between 24 hours and 72 hours in 20.38% of cases and more than 72 hours in 8.42% of cases. The high frequency of the patients in the first 24 hours would be explained by the spectacular aspect of the etiological circumstance and the seriousness of the wounded from the start.

As for the smaller influx of patients after the first 24 hours, this could be explained by the ignorance of the majority of the population about the severity and the possibility of secondary aggravation of cranio-encephalic lesions, due to a lack of resources, transport and the removal of specialized health structures. Those who assist the traumatized brain tend to send it first to the healer of the neighborhood before evacuating a second time to the nearest clinic and hospital. These same reasons explain the frequency of patients beyond 72 hours. This longer or shorter period would explain the importance of morbidity and mortality because serious head trauma in particular is a medical and surgical emergency because of the severity of the initial lesions and the possibility of very rapid secondary aggravation. Added to this is the possibility of a secondary cerebral aggression of systemic origin (ACSOS). All these signs mean that any delay in treatment can lead to a fatal outcome. This delay must be avoided by the orderly organization of rescue teams with sufficient technical equipment, good quality and qualified personnel to ensure quality pre-hospital care.

**Clinical aspects:** benign TBI account for more than 95% of head injuries in children in the USA [13] and severe TBI 1 to 2.1% of head injuries in the USA [14, 15]. In our study the majority of children (53.8%) had a mild TBI, 36.8% had moderate TBI and 9.4% had severe TBI. In Tunisia, Hassen *et al*. had observed 92.6% mild TBI, 2% moderate TBI and 5.4% severe TBI [6]. Of the 28 children with severe TBI, only 16 had intubation and assisted ventilation. This testifies to the insufficiency of places in intensive care unit. The 4 university hospital centers in Abidjan (Ivory Coast), reference centers have an intensive care unit each for a total of 30 respirators (Cocody 8, Yopougon 13, Treichville 5 and Angré 4) for 24 million Ivorians or about 0.166 units/1000000 inhabitants. This ratio is comparable to Uganda's 0.1 unit/1 million inhabitants [16] and well below what was observed in South Africa 8.9 units/100000 inhabitants, Sri Lanka 1.6 units/100000 inhabitants and United States of America 20 units per 100,000 inhabitants [17]. This lack of intensive care unit poses the problem of space availability. On the one hand, there is the problem of affordability to these intensive care unit because the necessary consumables must be prepaid by parents or relatives. On the other hand the cost of the surgical intervention also assured by parents or relatives. These situations constitute a barrier to the optimal care of children despite the availability of health care personnel.

**Figure 4:**
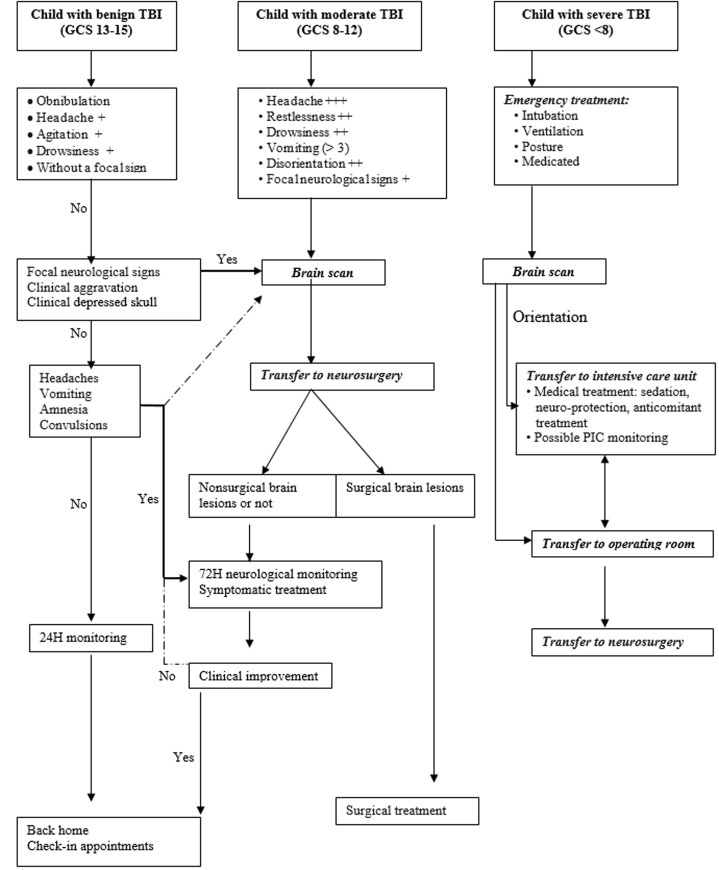
decisional algorithm in front of a child's TBI

**Paraclinical aspects:** the diagnosis of the TBI was essentially done at the brain scan. All our patients benefited from a brain scan. However, the accessibility of patients to this examination is reduced because of its cost which is 80 000 XOF or about 122 Euros. This reduced access to brain scans is another diagnostic barrier in our low-income country. The cerebral scan indication is formal for severe and moderate TBI but is controversial in case of mild TBI [18]. In our series, the indication was left to the discretion of the doctor who welcomed the child. Considering the financial cost of this examination in Ivory Coast (122 euros) and the daily cost of hospital surveillance varying between (6 euros and 10 euros) we propose a decision tree for the indication of cerebral scan for children of 2 to 15 years old with TBI (Figure 4). Children under the age of 2 should be scanned for TBI, regardless of severity.

**Therapeutic aspects:** we did not use the classic exploratory trepanation based on precise criteria. The use of a brain scan to diagnose cranio-encephalic lesions has made it possible to refine this surgical strategy for the 108 children operated in our series; or 36.9% of children who received surgical treatment. We had recourse to conventional treatments by the cranial component for the evacuations of extradural and subdural hematomas acute; trimming with duroplasty for cranio-cerebral wounds and emergence of embarrassments.

**Evolutionary aspects:** head injury in children is the leading cause of death in developed countries [14]. In the United States, the mortality rate of traumatic brain injury combined was about 6% [19]. In Tunisia, Hassen *et al*. reported mortality 2.1% in a hospital study [6]. In our study the overall mortality was 13.18% mainly due to the bad management of severe TBI whose mortality was 60.2% while the risk of death is estimated at 30% for severe TBI [20]. In Tunisia, Hassen *et al*. had observed a mortality of 37.5% for severe TBI [6] and Kpelao had found a mortality of 34.8% for severe TBI [8]. The lack of medicalized transport, the insufficiency and inaccessibility of intensive care units and imaging were factors aggravating the mortality observed in our study. The risk of death is 0.4 to 4% for moderate TBI and 0 to 2% for benign TBI [19], which corresponds to the respective mortality rates of 5.7% and 1.8% observed in our study. Postoperative mortality was nil.

## Conclusion

TBI was the leading cause of admission to pediatric emergencies at our institution. Road accidents were the main causes. The mortality of severe TBI was high because of the insufficiency and inaccessibility of intensive care and imaging units on the one hand, of medical transport and the absence of a social security system on the other hand. Care giver awareness in outlying hospitals is also needed to improve patient conditioning prior to transfer to pediatric emergencies. The management of severe head injuries requires the implementation of a health policy facilitating the accessibility of care and equipment of hospitals in the intensive care unit.

### What is known about this topic

- Traumatic brain injury in children represent the first cause of admission to the pediatric emergencies;
- Compared with their adult counterparts, children suffering head injury warrant particular concern given the developmental consequences of early brain damage;
- Traumatic brain injury in children constitute a real public health problem in developed countries and marked increase in underdeveloped countries.

### What this study adds

- Our experience on the management of traumatic brain injury in children with their particularities;
- We underline the difficulties of the adequate management of this affection in our country in particular and in general in the countries in the process of development;
- We have established a decisional algorithm, which we believe is relevant for care.
